# Supplementary material for: The effect of dose on the antimalarial efficacy of artesunate-mefloquine against Plasmodium falciparum malaria: a protocol for systematic review and individual patient data (IPD) meta-analysis
Source: BMJ Open. 2019 Jun 27;9(6):e027738. doi: 10.1136/bmjopen-2018-027738 (PMC6609048; doi:10.1136/bmjopen-2018-027738)
Supplement: Supplementary data [file bmjopen-2018-027738supp001.pdf]

Supplementary file: The effect of dose on the antimalarial efficacy of artesunate-mefloquine against *Plasmodium falciparum* malaria: a protocol for systematic review and individual patient data (IPD) meta-analysis

Rashid Mansoor[1,2], Prabin Dahal[1,2], Georgina S Humphreys[3], Philippe J Guerin[1,2], Elizabeth A Ashley[2,4], Kasia Stepniewska[1,2]*

[1]WorldWide Antimalarial Resistance Network (WWARN), Oxford, UK

[2]Centre for Tropical Medicine and Global Health, Nuffield Department of Clinical Medicine, University of Oxford, Oxford, UK

[3]Wellcome Trust, London, UK

[4]Myanmar Oxford Clinical Research Unit, Yangon, Myanmar

*Corresponding author

Dr. Kasia Stepniewska

Centre for Tropical Medicine and Global Health,

Nuffield Department of Medicine Research Building,

University of Oxford, Old Road Campus,

Roosevelt Drive, Oxford OX3 7FZ

Tel: +44-01865 612900

Email : kasia.stepniewska@wwarn.org

Literature Search Strategy

The following search terminologies were used for searching relevant publications in the given libraries

PubMed via PubMed

((("plasmodium falciparum"[MeSH Terms] OR ("plasmodium"[All Fields] AND "falciparum"[All Fields]) OR "plasmodium falciparum"[All Fields]) OR falciparum[All Fields]) OR ("malaria"[MeSH Terms] OR "malaria"[All Fields])) AND (((((artesunate-mefloquine[All Fields] OR artesunate/mefloquine[All Fields]) OR ASMQ[All Fields]) OR AS-MQ[All Fields]) OR AS/MQ[All Fields]) OR (("artesunate"[Supplementary Concept] OR "artesunate"[All Fields]) AND (("mefloquine"[MeSH Terms] OR "mefloquine"[All Fields]) OR MQ[All Fields])))

EMBASE via OVID

(plasmodium falciparum.mp. OR Plasmodium falciparum/ OR falciparum.mp. OR (falciparum.mp. AND (Plasmodium/ OR plasmodium.mp.)) OR malaria.mp. OR malaria/) AND (artesunatemefloquine.mp. OR ASMQ.mp. OR AS-MQ.mp. OR ((artesunate/ OR artesunate.mp.) AND (mefloquine/ OR mefloquine.mp. OR MQ.mp.))

BIOSIS via Web of Science

(TS=(plasmodium falciparum) OR (TS=(falciparum AND plasmodium)) OR TS=(falciparum) OR TS=(malaria)) AND TS=(artesunate-mefloquine) OR TS=(ASMQ) OR TS=(AS-MQ) OR (TS=(artesunate) AND TS=(mefloquine OR MQ))

Web of Science Core Collection via Web of Science

(TS=(plasmodium falciparum) OR (TS=(falciparum AND plasmodium)) OR TS=(falciparum) OR TS=(malaria)) AND TS=(artesunate-mefloquine) OR TS=(ASMQ) OR TS=(AS-MQ) OR (TS=(artesunate) AND TS=(mefloquine OR MQ))
