# Supplementary material for: The effect of dose on the antimalarial efficacy of artesunate-mefloquine against Plasmodium falciparum malaria: a protocol for systematic review and individual patient data (IPD) meta-analysis
Source: BMJ Open. 2019 Jun 27;9(6):e027738. doi: 10.1136/bmjopen-2018-027738 (PMC6609048; doi:10.1136/bmjopen-2018-027738)
Supplement: Author's manuscript [file bmjopen-2018-027738.draft_revisions.pdf]

BMJ Open is committed to open peer review. As part of this commitment we make the peer review history of every article we publish publicly available.

When an article is published we post the peer reviewers' comments and the authors' responses online. We also post the versions of the paper that were used during peer review. These are the versions that the peer review comments apply to.

The versions of the paper that follow are the versions that were submitted during the peer review process. They are not the versions of record or the final published versions. They should not be cited or distributed as the published version of this manuscript.

BMJ Open is an open access journal and the full, final, typeset and author-corrected version of record of the manuscript is available on our site with no access controls, subscription charges or pay-per-view fees (http://bmjopen.bmj.com).

If you have any questions on BMJ Open's open peer review process please email info.bmjopen@bmj.com

BMJ Open

The effect of dose on the antimalarial efficacy of artesunate-mefloquine against Plasmodium falciparum malaria: a protocol for systematic review and individual patient data (IPD) meta-analysis

Journal:	*BMJ Open*
Manuscript ID	bmjopen-2018-027738
Article Type:	Protocol
Date Submitted by the Author:	05-Nov-2018
Complete List of Authors:	Mansoor, Rashid; WorldWide Antimalarial Resistance Network (WWARN); University of Oxford, Centre for Tropical Medicine and Global Health, Nuffield Department of Medicine<br>Dahal, Prabin; WorldWide Antimalarial Resistance Network (WWARN); University of Oxford, Centre for Tropical Medicine and Global Health, Nuffield Department of Medicine<br>Humphreys, Georgina; WorldWide Antimalarial Resistance Network (WWARN); University of Oxford, Centre for Tropical Medicine and Global Health, Nuffield Department of Medicine<br>Burrow, Rebekah; WorldWide Antimalarial Resistance Network (WWARN); University of Oxford, Centre for Tropical Medicine and Global Health, Nuffield Department of Medicine<br>Guerin, Philippe; WorldWide Antimalarial Resistance Network (WWARN); University of Oxford, Centre for Tropical Medicine and Global Health, Nuffield Department of Medicine<br>Ashley, Elizabeth ; Myanmar-Oxford Clinical Research Unit (MOCRU), Yangon, Myanmar; University of Oxford, Centre for Tropical Medicine and Global Health, Nuffield Department of Medicine<br>Stepniewska, Kasia; WorldWide Antimalarial Resistance Network (WWARN); University of Oxford, Centre for Tropical Disease and Global Health, Nuffield Department of Medicine
Keywords:	malaria, Plasmodium falciparum, efficacy, IPD meta-analysis, mefloquine, artemisinin

SCHOLARONE™
Manuscripts

The effect of dose on the antimalarial efficacy of artesunate-mefloquine against *Plasmodium falciparum* malaria: a protocol for systematic review and individual patient data (IPD) meta-analysis

Rashid Mansoor[1,2], Prabin Dahal[1,2], Georgina S Humphreys[1,2], Rebekah Burrow[1,2], Philippe J Guerin[1,2], Elizabeth A Ashley[2,3], Kasia Stepniewska[1,2]*

[1]WorldWide Antimalarial Resistance Network (WWARN), Oxford, UK

[2]Centre for Tropical Medicine and Global Health, Nuffield Department of Clinical Medicine, University of Oxford, Oxford, UK

[3]Myanmar Oxford Clinical Research Unit, Yangon, Myanmar

Running title: dose effect of artesunate mefloquine

*Corresponding author

Dr. Kasia Stepniewska: kasia.stepniewska@wwarn.org

Address: Centre for Tropical Medicine and Global Health, Nuffield Department of Medicine Research Building, University of Oxford, Old Road Campus, Roosevelt Drive, Oxford OX3 7FZ

Tel: +44-01865 612900

Email: rashid.mansoor@wwarn.org (RM); prabin.dahal@wwarn.org (PD); georgina.humphreys@wwarn.org (GSH); rebekah.burrow@wwarn.org (RB); philippe.guerin@wwarn.org (PJG); liz@tropmedres.ac (EAA); kasia.stepniewska@wwarn.org (KS)

Word count 3776

For peer review only

For peer review only - http://bmjopen.bmj.com/site/about/guidelines.xhtml

Abstract

Introduction: Antimalarial posology based on weight-bands leave patients at the margins vulnerable to receiving either lower or higher weight-adjusted (mg/kg) dosages. This study aims to describe the distribution of artesunate and mefloquine dosage administered in patients with uncomplicated *P. falciparum* malaria treated with an artesunate-mefloquine (AS-MQ) regimen. Relationship between mg/kg dose and therapeutic outcomes will be assessed through a one-stage individual participant data (IPD) meta-analysis (MA).

Methods and analysis: Therapeutic efficacy studies with the AS-MQ regimen will be identified by searching the following databases: PUBMED, EMBASE, and Web of Science. The corresponding authors of the relevant studies will be requested to share the IPD for the purpose of this meta-analysis to a secured repository. All available studies will be standardised using a common data management protocol and pooled into a single database. The relationship between mg/kg dosage and treatment failures will be assessed using a Cox regression model with study sites considered as a shared frailty term. Safety parameters will be explored where available.

Ethics and Dissemination: This IPD meta-analysis met the criteria for waiver of ethical review as defined by the Oxford Tropical Research Ethics Committee (OxTREC) as the research consists of secondary analysis of existing anonymous data. The results of this analysis will be disseminated at conferences, WWARN website and any peer-reviewed publication arising will be made open-source.

PROSPERO Registration: CRD42018103776

Keywords: malaria, *Plasmodium falciparum*, efficacy, IPD meta-analysis, mefloquine, artemisinin, artemisinin combination therapy

For peer review only - http://bmjopen.bmj.com/site/about/guidelines.xhtml

Strengths and limitations of this study

- With the exception of recent studies in Southeast Asia, the regimen artesunate-mefloquine has consistently demonstrated an efficacy greater than 95% and treatment failures in any single antimalarial study are few. This IPD-MA will allow a robust exploration of host, parasite and drug factors associated with therapeutic outcomes, which otherwise would not be possible.
- The proposed IPD-MA will allow exploration of variations in the weight-adjusted dosage received by patients, which is not possible with aggregate data meta-analysis.
- The IPD-MA will be carried out as a study group under the auspices of the WorldWide Antimalarial Resistance Network (WWARN), which has championed responsible data sharing and advocates translational research.
- A limitation of this analysis will be heterogeneity between studies included in terms of design, patient population and the susceptibility of the parasites against the drug regimen.

For peer review only - http://bmjopen.bmj.com/site/about/guidelines.xhtml

Introduction

The combination artesunate-mefloquine (AS-MQ) was the first antimalarial regimen developed as an Artemisinin Combination Therapy (ACT) when mefloquine resistance became rampant along the Thai-Myanmar border in the early 1990s [1]. The efficacy of a combination regimen (artemisinin derivative + partner component) depends upon the ability of the partner component to mop up the residual parasites leftover after the initial and highly potent anti-parasitic activity of the artemisinin derivatives. This requires the dosage of the partner drug to be sufficient to ensure that blood concentrations exceed the Minimum Inhibitory Concentration (MIC) of the parasites until all the parasites have been killed. Manufacturers' recommendations regarding antimalarial posology are often pragmatic and the dose is administered based upon weight "banding". This approach inevitably results in some patients at the band margins receiving either lower or higher dosages when adjusted for body weight (Figure 1). Young children are particularly vulnerable to extreme total dosages especially when drug administration is based on tablets rather than paediatric formulations or suspensions. This may lead to sub-therapeutic drug concentrations in the blood plasma and such under-exposure has been related to poorer therapeutic response for some of the widely used ACTs [2–4].

Until recently ACTs have consistently demonstrated an efficacy greater than 95% and treatment failures in any single antimalarial study are few thus limiting the ability to draw inferences regarding putative factors associated with therapeutic outcomes. Individual participant data (IPD) meta-analysis (MA) is being used increasingly to explore some of the putative factors which otherwise would not be possible through aggregate data meta-analysis[5]. Such a IPD-MA approach has been used to assess the dose-response relationships for the ACT regimens of artesunate-amodiaquine, artemether-lumefantrine and dihydroartemisinin-piperaquine [2,3,6]. These studies have demonstrated that the drug formulation (fixed vs loose) and under-exposure in the paediatric population due to weight-banding are deterministic of

For peer review only - http://bmjopen.bmj.com/site/about/guidelines.xhtml

poorer therapeutic outcomes. A thorough evaluation of the dose-response relationship for the regimen AS-MQ is lacking and this IPD-MA aims to address this research gap.

Objectives

The overall aim of this study is to determine the mg/kg dosing range of artesunate (AS) and mefloquine (MQ) adopted in clinical trials and to investigate the effects of mg/kg dosing on clinical outcome.

The specific objectives are:

- To investigate the effects of mefloquine and artesunate mg/kg dosing on early and late clinical outcomes (treatment success or failure)
- To investigate the tolerability of AS-MQ across different study sites, population and age groups

For peer review only

For peer review only - http://bmjopen.bmj.com/site/about/guidelines.xhtml

Methods and analyses

Criteria for study eligibility

Studies identified will be deemed eligible for the purpose of this analysis if they meet the following criteria:

- Prospective clinical efficacy study of uncomplicated *P. falciparum* (either alone or mixed infections)
- Assessing the efficacy of a fixed-dose AS-MQ combination, either as single tablet type, or co-blister pack of more than one tablet type, or assessing the efficacy of a loose combination of AS-MQ
- Where AS was given over three days (with any number of doses per day) with a target total dose of 6-30mg/kg
- Where MQ was given over 1-3 days, on any of days 1-3 (with any number of doses per day) with a target total dose of 15-33mg/kg
- Where all AS and MQ were administered orally
- With a minimum of 28 days follow up
- With genotyping performed for late parasite recurrence
- With individual patient data on dosage of mefloquine received (actual or per protocol) by patients (dosage per tablets, number of tablets given per dose and duration of treatment)

Criteria for study exclusion

• Where other antimalarial drugs were given in addition to the initial ASMQ treatment regimen, except for a single dose of primaquine of 0.25mg/kg in the first 3 days

For peer review only - http://bmjopen.bmj.com/site/about/guidelines.xhtml

Types of study participants

Patients with uncomplicated *P. falciparum* malaria will be included in this IPD-MA. The following patients will be excluded from the analysis:

i. Severe *P. falciparum* malaria
ii. Pregnant women

Types of intervention/exposure and controls

- Fixed dose combination of AS-MQ, either as single tablet type, or co-blister pack of more than one tablet type, or loose combination of AS-MQ. AS given over three days (with any number of doses per day) with a target total dose of 6-30mg/kg. MQ given over 1-3 days, on any of days 1-3 (with any number of doses per day) with a target total dose of 15-33mg/kg

Types of outcomes

i. Parasitological and clinical efficacy
ii. Adverse events

Information sources and search strategy

We will carry out a systematic review and search PubMed, EMBASE, and Web of Science to identify publications with artesunate-mefloquine between Jan 1990 to July 2018, the full search terms are available from the PROSPERO registration (CRD42018103776). Any important protocol amendments will be documented in the PROSPERO registration. Studies will be included regardless of language and publication status. Study screenings will be carried by two independent reviewers who will screen title, abstract, full text as necessary. The following studies will be excluded: animal models (e.g. mouse malarias *P. berghei, P. chabaudi*), publications only including severe malaria, studies with follow up <28 days, data previously

For peer review only

For peer review only - http://bmjopen.bmj.com/site/about/guidelines.xhtml

141 included in another published study, prophylaxis or mass drug administration studies, studies in healthy volunteers/challenge studies, or studies in asymptomatic patients or pregnant women.

143 ***Data acquisition and data management***

144 **Collating IPD**

145 Principal investigators of the published (or unpublished) studies identified from the literature search will be invited to share IPD. At least three emails will be sent out in case of non-response. Researchers agreeing to the terms and conditions of the submission will be requested to upload anonymised IPD to the WWARN repository through a secure web portal. Figure 2 shows the process map which depicts the different phases of data procurement; data acquisition from the contributors, data standardisation, and their subsequent re-use in IPD meta-analysis.

151 Data will be fully anonymised and handled in compliance with the UK Data Protection Act to protect personal information and patient privacy. Original data will be stored on a secure server hosted by the University of Oxford.

154 **Data management**

155 Raw data from individual studies will be standardised using an open and transparent data management and statistical analysis protocol[7]. Investigators will be further contacted for validation or clarification, if required, and individual study protocols will be requested. On standardisation, the data will be stored in a relational database of several tables containing information on drug regimen, parasitological, clinical, and haematological assessments, genotyping and therapeutic outcomes; all linked by a unique patient identifier.

161 **Statistical analysis plan**

162 ***Study Population***

163 The following patients will be included in the analysis:

For peer review only - http://bmjopen.bmj.com/site/about/guidelines.xhtml

i. Information is available on drug dosage, either as exact number of tablets received, exact mg/kg dose received or number of tablets planned per protocol

ii. Date of the last day of follow-up or length of follow-up

The following patients will be excluded from the analysis:

i. Received other antimalarial drugs during follow-up before recorded *P. falciparum* treatment failure

ii. Results of genotyping performed for late parasitological outcome are not available

iii. Missing confirmation of *P. falciparum* infection on enrolment

iv. Missing age or weight or gender

v. Other deviations, as defined in the data management plan[7] :

(a) Haemoglobin < 5 g/dL on day 0

(b) Haematocrit < 15% on day 0

Outcomes

Primary: PCR-corrected *P. falciparum* recrudescence

Secondary: PCR-corrected *P. falciparum* reinfection

PCR-uncorrected *P. falciparum* recurrence

Early parasitological responses on days 1, 2 and 3

Gametocyte carriage within 14 days of treatment initiation in patients without gametocytaemia at enrolment

Anaemia status on day 7

Adverse symptoms developed after the drug administration

For peer review only - http://bmjopen.bmj.com/site/about/guidelines.xhtml

For peer review only

The primary endpoint for this IPD-MA is Polymerase Chain Reaction (PCR) genotyping corrected risk of *P. falciparum* recrudescence (treatment failure) on day 42. In the analysis of the primary endpoint, patients in whom new infections are observed during the study follow-up, or those who are lost to follow-up will be censored; the former on the day new infection was observed and the latter on their last recorded visit day. For the analysis of PCR corrected new infections, patients with recrudescence and those who are lost to follow-up will be censored. Further definitions of status and other censorship are detailed in the Clinical Module DMSAP [7].

Acute drug vomiting within an hour of treatment administration, general vomiting within 7 days of treatment initiation, diarrhoea within 7 days of treatment initiation, and, neuropsychiatric adverse events (where available) are secondary endpoints.

For peer review only

For peer review only - http://bmjopen.bmj.com/site/about/guidelines.xhtml

Variables and definition

Artesunate and mefloquine doses received will be calculated from the number of tablets administered to each patient daily. If the actual number of tablets received is not recorded, the total dose in mg or mg/kg recorded as administered to each patient will be used. If none of these are available, administration as per protocol will be assumed. The current recommended daily mefloquine dose is 8.3 (range: 5 - 11) mg/kg [8]. A patient will be classified as under-dosed if the 3-day total mg/kg mefloquine dose is less than 15 mg/kg.

Nutritional status in children under 5 years of age will be assessed using standardised age, weight, height and gender specific growth reference standards according to the WHO 2006 recommendations using igrowup Stata package [9]. Anthropometric indicators include weight-for-age (WAZ), height-for-age (HAZ), and weight-for-height (WHZ). The nutritional status of a child will be given as a Z-score and classified as stunted, underweight or wasted as defined in the WHO guidelines.

The falciparum malaria transmission intensity of the study sites will be assessed using the prevalence estimates generated by the Malaria Atlas Project (MAP) based on the latitude, longitude and the year the study was conducted [10,11].

Anaemia will be defined as haemoglobin (Hb) < 10 g/dL or haematocrit (Ht) < 30%. Severe anaemia is defined as haemoglobin < 7 g/dL or haematocrit < 20%. Fever will be defined as body temperature > 37.5 degrees Celsius.

Parasite resistance status will be defined (data permitting) for each patient from South-East Asia region based on the reported prevalence of mutations of molecular markers (pfmdr1, kelch13) or the distribution of parasite clearance half-life for their study site and year of

For peer review only - http://bmjopen.bmj.com/site/about/guidelines.xhtml

admission [12,13]. For other locations, we will assume that parasites are sensitive to the ASMQ regimen.

Descriptive summary

Summary of the studies

Summary of included studies will be presented with respect to: study location, years of study, study population, duration of follow-up, AS-MQ drug formulation, methodology for parasite quantification, methodology for PCR genotyping, supervision of drug administration. PCR-corrected and uncorrected outcomes will be used to compute the Kaplan-Meier (K-M) estimates using the censoring rules outlined above. The K-M estimates will be presented graphically together with the number of patients in the risk set.

Summary of the patients

Summary of baseline characteristics of the patients included in the analysis will be presented for each study, by region and in overall. The following baseline characteristics of patients will be presented: age; weight, parasitaemia on enrolment; presence of fever (body temperature > 37.5 degrees Celsius); haemoglobin (or haematocrit); anaemia (Hb < 10 g/dL) or severe anaemia (Hb < 7 g/dL), gametocytes on presentation, description of infection (*P. falciparum* or mixed infections), total mg/kg dose for each drug component, dosing strategies (age-based, weight based etc.), dose formulation (fixed or loose etc.), and supervision of drug administration. The number of available patients will be summarised for all variables, proportion will be used for categorical or binary variables, and mean and standard deviation (or median and range) will be used for continuous variables.

Analysis of the primary endpoint

The efficacy estimates for each of the studies will be summarised using the Kaplan-Meier (K-M) method.

For peer review only - http://bmjopen.bmj.com/site/about/guidelines.xhtml

Cox regression analysis will be carried out to identify the predictors associated with parasitic recrudescence using a one-stage IPD-MA. Random effects in the form of shared gamma frailty parameters will be used to adjust for study-site effect and account for unobserved statistical heterogeneity [14]. Schoenfeld residuals against transformed time will be used to determine if the assumption of proportional hazard is met. Cox-Snell residuals will be examined to determine the appropriateness of model fit. Martingale residuals will be used to assess the functional form of the covariates. Potential non-linear relationships between continuous variable and the treatment outcomes will be investigated using multivariable fractional polynomials (FP)[15]. If the assumption of proportional hazards is not satisfied, alternative approaches such as piecewise proportional hazards model, interaction with time, stratifying by the variable for which the assumption isn't satisfied or flexible parametric models will be considered. Variable selection process will follow a procedure described below.

Analyses of secondary endpoints

***P. falciparum* new infection:** The analysis of new infections will be similar to the analysis of the primary endpoint.

Parasite clearance: Early parasitological responses will be assessed by the parasite positivity rate (PPR), which is the proportion of patients remaining parasitaemic on days 1, 2 and 3 post treatment administration[16]. The relationship between mg/kg dosage of the artesunate and mefloquine on early parasitological responses will be explored using a logistic regression model with study sites fitted as random effect. Variable selection and additional sensitivity analyses will follow the plan as outlined for the primary endpoint.

Gametocyte carriage: Gametocyte carriage during the study follow-up will be stratified by the gametocytaemia status at baseline. For those with documented gametocytaemia at enrolment, proportion of patients in whom gametocyte has cleared will be reported. For those

For peer review only - http://bmjopen.bmj.com/site/about/guidelines.xhtml

without gametocytes on enrolment, the proportion of patients in whom gametocytes have evolved will be presented. The relationship between mg/kg dosage of the artesunate and mefloquine on gametocyte endpoints will be explored using a logistic regression model with study sites fitted as random effect. Variable selection and additional sensitivity analyses will follow the plan as outlined for the primary endpoint.

Haematological insult: Anaemia during the study follow-up will be stratified by the anaemia status at baseline. For those who are anaemic at enrolment (, the proportion of patients who have recovered will be reported. For those who are not anaemic at enrolment, the proportion of patients whom are subsequently anaemic will be presented. The relationship between mg/kg dosage of the artesunate and mefloquine on anaemia endpoints will be explored using a logistic regression model with study sites fitted as random effect. Variable selection and additional sensitivity analyses will follow the plan as outlined for the primary endpoint.

Safety endpoints: The proportion of patients with acute drug vomiting, vomiting and diarrhoea, neuropsychiatric adverse events within a week of treatment initiation will be reported. The relationship between the mg/kg mefloquine dose and safety endpoints will be evaluated using a logistic regression model with study sites fitted as a random effect, if data permits. Variable selection and additional sensitivity analyses will follow the plan as outlined for the primary endpoint.

For peer review only

For peer review only - http://bmjopen.bmj.com/site/about/guidelines.xhtml

Variable selection strategy

The following covariates will be examined: age, sex, weight, baseline parasitaemia (except for new infection analysis), weight-for-age z-score (WAZ), underweight for age termed underweight (defined as WAZ < -2), haemoglobin, gametocytes on presentation (except for new infection analysis), past history of malaria (if available); description of infection: mixed species infections (except for new infection analysis), presence of markers of drug resistance e.g. kelch13 mutations or *pfmdr1* amplification (if available), details of treatment received: total mg/kg dose of AS and MQ, regimen, drug supervision, and vomiting of medication. Year of enrolment will also be included to account for changes in parasite susceptibility over time.

A general strategy recommended by Collet (2015)[17] will be followed for the construction of multivariable regression model:

i) All possible risk factors will be examined in a univariable analysis. The log-likelihood estimates ($-2 \times Log\hat{L}$) will be compared against the null model to assess if any of the variables reduce its value at 5% level of statistical significance.

ii) All the variables identified in step (i) will be fitted together in one model and variables which are not significant in the presence of other variables based on the results of the Wald test will be identified.

iii) A likelihood ratio test will be used to assess the impact of omitting variables identified in step (ii). If the omitted variable does not significantly impact the model log-likelihood, then they will be dropped. Only those variables which lead to significant change in log-likelihood are retained.

iv) All variables excluded from step (i) will be added to the model identified in step (iii) one by one to check if they provide any improvement to the model.

v) A final check of the model identified in step (iv) will be carried out to ensure that none of the variables in the model can be omitted without significantly increasing the model

For peer review only - http://bmjopen.bmj.com/site/about/guidelines.xhtml

log-likelihood, and none of the excluded variables significantly reduces the model log-likelihood.

Comparison of likelihood between nested models will be conducted using Likelihood Ratio Test (LRT). Akaike's Information Criterion (AIC) will be used to compare non-nested models. Treatment dosage, drug formulation, and baseline parasitaemia will be included in the multivariable model as *a priori* forced variables regardless of their statistical significance. Variables with more than 50% observations missing will not be included in multivariable analysis. Interactions will be assessed between dosing and the following variables: region, age group, transmission intensity, hyperparasitaemia (parasitaemia>100,000 parasites per microlitre), date of enrollment.

Assessment of statistical heterogeneity

The multilevel logistic or Cox models will be used for explaining study-site heterogeneity. Heterogeneity across study sites will be assessed by the variance of the shared frailty term estimated in the random effect Cox model or variance of the random intercepts in logistic regression. Additionally, intra-class correlation in logistic regression model will be reported.

Subgroup analyses

Analyses will be conducted by geographical region, drug regimen and resistance status if data permit.

Sensitivity analyses

A model will be refitted with each study's data excluded, one at a time, and a coefficient of variation around the parameter estimates will be calculated. This would identify any influential studies, that is, studies with unusual results (due to variations in methodology, patient population, and so on) that affect the overall pooled analysis findings. To assess the impact of missing data (covariates, PCR genotyping results), sensitivity analysis will be performed to see

For peer review only - http://bmjopen.bmj.com/site/about/guidelines.xhtml

333 if our main conclusion is affected or not by the exclusion of patients with missing data. Multiple Imputation (MI) will be used for handling missing data for missing covariates and missing outcomes. MI will be carried out for covariates with missing proportion less than 50%. [18]

Quality assessment/risk of bias assessment in studies included

Two reviewers will independently assess risk of bias. The risk of bias within and across the studies included in the analysis will be carried out using the GRADE guidelines[19]. Cochrane risk of bias tool 2.0 will be used to assess risk of bias in individual randomised controlled trails. Publication bias will assessed by a funnel plot.

Assessment of risk of potential bias in missing studies

Despite best possible efforts, it is anticipated that raw data from all the identified studies will not be available. Risk of potential bias in these studies will be assessed using a two-stage IPD-MA for the reported efficacy outcomes.

For peer review only

For peer review only - http://bmjopen.bmj.com/site/about/guidelines.xhtml

Further development of statistical analysis plan

The main analysis is planned as described above. Modification or additional analyses may be required as the data collection progresses. An updated statistical analysis plan will be available on the WWARN study group website[20].

Software

All statistical analyses will be carried out using R (The R Foundation for Statistical Computing) or Stata (4905 Lakeway Drive, College Station, Texas 77845 USA). Using alternative statistical software will not require amendment of this SAP.

Ethics and dissemination

This IPD meta-analysis met the criteria for waiver of ethical review as defined by the Oxford Tropical Research Ethics Committee (OxTREC) as the research consists of secondary analysis of existing anonymous data[21]. All studies included in this analysis will have received local ethical approvals and our pooled IPD meta-analysis will be addressing scientific questions that are very similar to the original research questions.

Findings will be reported following the PRISMA-IPD guidelines[22] at open access peer-reviewed journals. This systematic literature review and IPD meta-analysis is registered to PROSPERO (CRD42018103776) and this protocol has been reported following the PRISMA-P guidelines[23]. Any publications based on the findings of this IPD meta-analysis will be in accordance with the guidelines of the International Committee of Medical Journal Editors.

For peer review only

For peer review only - http://bmjopen.bmj.com/site/about/guidelines.xhtml

Discussion

Large scale deployment of highly efficacious ACT regimens such as artesunate-mefloquine has been the cornerstone of global malaria control for over a decade and this has contributed to the global reduction of mortality and morbidity associated with malaria [11]. Maintaining these gains is highly dependent on efficient health systems, sustainable global funding and the current status of antimalarial drug resistance. The 2017 WHO report found that globally the number of malaria cases has stopped dropping and mortality has crept up compared to 2016, suggesting that the recent public health gains remain fragile [24,25]. To make things worse, a health calamity is looming large due to the emergence of resistance to artemisinins in South-East Asia which is threatening to reverse the remarkable progress achieved over the past decade [26]. In the absence of an alternative treatment class to replace the ACTs as first line therapy, preserving the currently available drugs remains the top-most priority and this requires the highest form of evidence regarding the susceptibility of the parasites against the antimalarial drugs. Artesunate-mefloquine retains extremely high efficacy in most locations except Thailand, and in any given trial, only few treatment failures have been observed which limits the utility of any single study in answering questions regarding the dose-response relationship. Individual participant data (IPD) meta-analysis provides an alternative strategy.

IPD meta-analysis is now considered the gold-standard for evidence synthesis and allows exploration of different risk factors which otherwise would not be possible through the aggregate data meta-analysis [5]. This IPD-MA is designed to explore the variability in drug dosage administered in patients with uncomplicated *P. falciparum* malaria, treated with artesunate-mefloquine (AS-MQ). The WHO-recommended AS-MQ regimen is administered as a three day course, with a total of 12 mg/kg artesunate and 25 mg/kg of mefloquine split over three days. Due to the poor tolerability of high dose mefloquine, the dose of mefloquine is usually divided into either two doses (15 and 10 mg/kg), or three as a fixed dose combination

For peer review only - http://bmjopen.bmj.com/site/about/guidelines.xhtml

(8mg/kg/day). The fixed dose combination has been shown to provide better efficacy and improve treatment adherence for artesunate-amodiaquine [6]. Such a comparison is yet to be made for the AS-MQ regimen, and in this IPD-MA we propose to compare the fixed and loose formulations of the regimen with regards to the drug dosing, tolerability, efficacy and practicality of the dose banding.

In conclusion, this pooled analysis will provide critical information regarding the relationship between drug dosage and parasitological responses post-treatment with artesunate-mefloquine. The assessment of the host, parasite and drug determinants that influence the treatment response can provide evidence-based guidance for monitoring the early signs of artemisinin resistance and effective case management that will be critical in optimising malaria control and containment efforts.

For peer review only

For peer review only - http://bmjopen.bmj.com/site/about/guidelines.xhtml

Declarations

Author contributions

Conceived the idea and wrote the first draft of the protocol: RM, PD, PG, EAA, and KS

Systematic review of all published antimalarial studies: RB and GSH

Data acquisition and standardisation: GSH, RB, EAA, KS

All authors read and approved the submission of the final draft of the study protocol.

Funding

The WorldWide Antimalarial Resistance Network (RM, PD, GSH, RB, PJG, and KS) is funded by a Bill and Melinda Gates Foundation grant and the ExxonMobil Foundation. The funders did not participate in the study development, the writing of the paper, decision to publish, or preparation of the manuscript.

Competing interests

The authors declare that they have no competing interests.

Ethics approval

This individual patient data meta-analysis met the criteria for waiver of ethical review as defined by the Oxford Tropical Research Ethics Committee (OxTREC) since the research consists of secondary analysis of existing anonymous data. Each study included in the analysis will have received local ethics approvals.

Acknowledgements

We would like to thank Dr. Makoto Saito for his several helpful suggestions.

For peer review only - http://bmjopen.bmj.com/site/about/guidelines.xhtml

List of Figures

Figure 1: Mg/kg dose variations for mefloquine

Legend: The WHO recommendation on the 3-day fixed dose AS-MQ regimen is to administer once daily 50 mg of MQ for those weighing 5-8 kg, 100 mg for 9-17 kg, 200 mg for 18-29 kg and 400mg for those who weigh ≥ 30 kg. The horizontal dotted line represents the target MQ dose of 25 mg/kg. Since the dosing is based on weight-bands, this leads to the saw-tooth dosing curves.

Figure 2: Data acquisition and standardisation process utilizing the WWARN database

Legend: Individual participant data is shared by researchers to the WWARN secured portal (left panel). The studies are standardised using a common WWARN protocol and the tables of clinical, parasitological and drug measurements are stored in a secured repository with relational database (middle panel). On standardisation, the dataset is shared back with the study investigator, and subsequently used in meta-analyses to answer questions of public health importance (right panel).

For peer review only

References

1. Nosten F, Luxemburger C, ter Kuile FO,. *et al.* Treatment of multidrug-resistant Plasmodium falciparum malaria with 3- day artesunate-mefloquine combination. *J. Infect. Dis.* 1994;**170:**971–7.

2. The WorldWide Antimalarial Resistance Network (WWARN) DP Study Group. The Effect of Dosing Regimens on the Antimalarial Efficacy of Dihydroartemisinin-Piperaquine: A Pooled Analysis of Individual Patient Data. *PLoS Med.* 2013;**10:**1–17.

3. WorldWide Antimalarial Resistance Network (WWARN) Lumefantrine PK/PD Study Group. Artemether-lumefantrine treatment of uncomplicated Plasmodium falciparum malaria: a systematic review and meta-analysis of day 7 lumefantrine concentrations and therapeutic response using individual patient data. *BMC Med.* 2015;**13:**227.

4. Hoglund RM, Workman L, Edstein MD, *et al.* Population Pharmacokinetic Properties of Piperaquine in Falciparum Malaria: An Individual Participant Data Meta-Analysis. *PLoS Med.* 2017;**14**:1–23.

5. Riley RD, Lambert PC & Abo-Zaid G. Meta-analysis of individual participant data: Rationale, conduct, and reporting. *BMJ* 2010;**340:**521–5.

6. The WorldWide Antimalarial Resistance Network (WWARN) AS-AQ Study Group. The effect of dosing strategies on the therapeutic efficacy of artesunate-amodiaquine for uncomplicated malaria: a meta-analysis of individual patient data. *BMC Med.* 2015;**13:**66.

7. WorldWide Antimalarial Resistance Network (WWARN). Clinical Module: Data Management and Statistical Analysis Plan Version 1.2. 2012. Available at: http://www.wwarn.org/sites/default/files/ClinicalDMSAP.pdf. (Accessed: 24th

For peer review only - http://bmjopen.bmj.com/site/about/guidelines.xhtml

460 September 2018)

461 8. World Health Organization. Guidelines for the treatment of malaria: Third edition. Geneva: World Health Organization 2015.

463 9. WHO Multicentre Growth Reference Study Group. WHO Child Growth Standards: Length/height-for-age, weight-for-age, weight-for-length, weight-for-height and body mass index-for-age: Methods and development. Geneva: World Health Organization 2006.

467 10. Gething PW, Patil AP, Smith DL, *et al.* A new world malaria map: Plasmodium falciparum endemicity in 2010. *Malar. J.* 2011;**10:**378.

469 11. Bhatt S, Weiss DJ, Cameron E, *et al.* The effect of malaria control on *Plasmodium falciparum* in Africa between 2000 and 2015. *Nature* 2015; 526(7572):207-11.

471 12. WWARN Parasite Clearance Study Group. Baseline data of parasite clearance in patients with falciparum malaria treated with an artemisinin derivative: an individual patient data meta-analysis. *Malar. J.* 2015;**14:**359.

474 13. WorldWide Antimalarial Resistance Network (WWARN). WWARN Molecular Surveyor. 2018. Available at: http://www.wwarn.org/molecularsurveyor/. (Accessed: 22nd October 2018)

477 14. Glidden DV & Vittinghoff E. Modelling clustered survival data from multicentre clinical trials. *Stat. Med.* 2004;**23:**369–88.

479 15. Royston P & Sauerbrei W. Fractional Polynomials for One Variable. in Royston P. & Sauerbrei W. Multivariable Model-Building: A Pragmatic Approach to Regression Analysis based on Fractional Polynomials for Modelling Continuous Variables. Chichester, UK: John Wiley & Sons, Ltd 2008: 71-98.16. WWARN ACT Africa

For peer review only - http://bmjopen.bmj.com/site/about/guidelines.xhtml

For peer review only

Baseline Study Group. Clinical determinants of early parasitological response to ACTs in African patients with uncomplicated falciparum malaria: a literature review and meta-analysis of individual patient data. *BMC Med.* 2015;**13:**212.

17. Collett D. Strategy for model selection. in Collett D. Modelling survival data in medical research. New York, US: CRC Press 2015:83-7.

18. White IR., Royston P & Wood AM. Multiple imputation using chained equations: Issues and guidance for practice. *Stat. Med.* 2011;**30:**377–99.

19. Guyatt G, Oxman AD, Akl EA, *et al.* GRADE guidelines: 1. Introduction - GRADE evidence profiles and summary of findings tables. *J. Clin. Epidemiol.* 2011;64(4):383–94.

20. WorldWide Antimalarial Resistance Network (WWARN). The WWARN AS-MQ Dose Impact Study Group. 2018. Available at: http://www.wwarn.org/working-together/study-groups/mq-dose-impact-study-group. (Accessed: 24th September 2018)

21. Oxford Tropical Research Ethics Committee. 2018. Available at: https://researchsupport.admin.ox.ac.uk/governance/ethics/committees/oxtrec. (Accessed: 24th September 2018)

22. Stewart LA, Clarke M, Rovers M, *et al.* Preferred reporting items for a systematic review and meta-analysis of individual participant data: The PRISMA-IPD statement. *J. Am. Med. Assoc.* 2015;**313:**1657–65.

23. Shamseer L, Moher D, Clarke M, *et al.* Preferred reporting items for systematic review and meta-analysis protocols (PRISMA-P) 2015: Elaboration and explanation. *BMJ* 2015;**349:**1–25.

24. World Health Organization. World Malaria Report 2017. Geneva: World Health

For peer review only

For peer review only - http://bmjopen.bmj.com/site/about/guidelines.xhtml

506 Organization 2017.

507 25. World Health Organization. World Malaria Report 2016. Geneva: World Health
508 Organization 2016.

509 26. Ashley EA, Dhorda M, Fairhurst RM, *et al.* Spread of Artemisinin Resistance in
510 Plasmodium falciparum Malaria. *N. Engl. J. Med.* 2014;**371:**411–23.

For peer review only

For peer review only - http://bmjopen.bmj.com/site/about/guidelines.xhtml

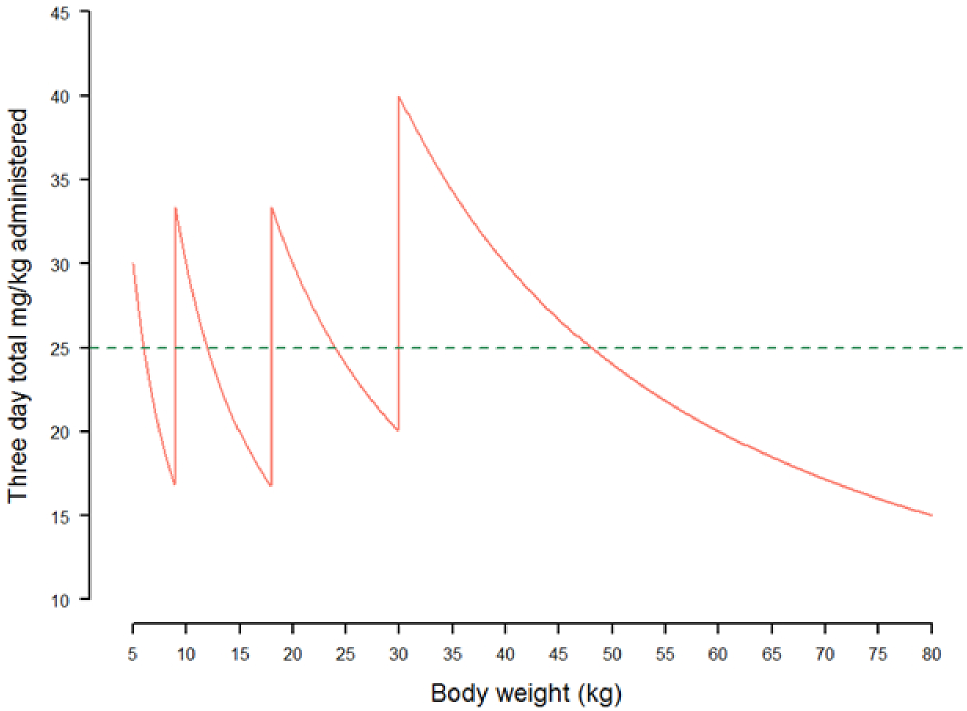

Figure 1: Mg/kg dose variations for mefloquine

168x130mm (96 x 96 DPI)

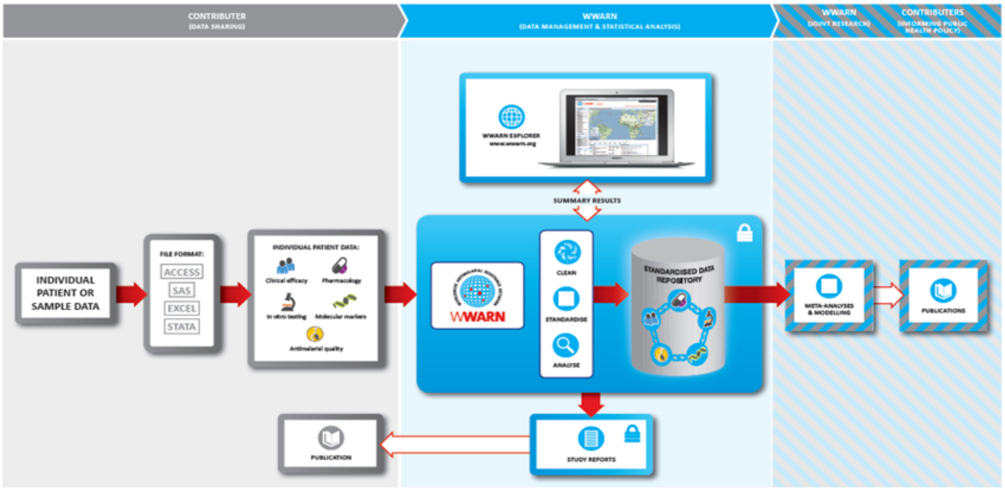

Figure 2: Data acquisition and standardisation process utilizing the WWARN database

223x108mm (96 x 96 DPI)

PRISMA-P 2015 Checklist

This checklist has been adapted for use with protocol submissions to *Systematic Reviews* from Table 3 in Moher D et al: Preferred reporting items for systematic review and meta-analysis protocols (PRISMA-P) 2015 statement. *Systematic Reviews* 2015 **4**:1

Section/topic	#	Checklist item	Information reported		Line number(s)
			Yes	No	
**ADMINISTRATIVE INFORMATION**					
**Title**					
Identification	1a	Identify the report as a protocol of a systematic review	X		2-3
Update	1b	If the protocol is for an update of a previous systematic review, identify as such		X	NA
**Registration**	2	If registered, provide the name of the registry (e.g., PROSPERO) and registration number in the Abstract	X		44
**Authors**					
Contact	3a	Provide name, institutional affiliation, and e-mail address of all protocol authors; provide physical mailing address of corresponding author	X		5-22
Contributions	3b	Describe contributions of protocol authors and identify the guarantor of the review	X		402- 406
**Amendments**	4	If the protocol represents an amendment of a previously completed or published protocol, identify as such and list changes; otherwise, state plan for documenting important protocol amendments		X	NA
**Support**					
Sources	5a	Indicate sources of financial or other support for the review	X		407- 411
Sponsor	5b	Provide name for the review funder and/or sponsor	X		409
Role of sponsor/funder	5c	Describe roles of funder(s), sponsor(s), and/or institution(s), if any, in developing the protocol	X		409-411
**INTRODUCTION**					
**Rationale**	6	Describe the rationale for the review in the context of what is already known	X		61- 86
**Objectives**	7	Provide an explicit statement of the question(s) the review will address with reference to participants, interventions, comparators, and outcomes (PICO)	X		87- 95
**METHODS**					

For peer review only

BioMed Central
The Open Access Publisher

Section/topic	#	Checklist item	Information reported Yes	Information reported No	Line number(s)
**Eligibility criteria**	8	Specify the study characteristics (e.g., PICO, study design, setting, time frame) and report characteristics (e.g., years considered, language, publication status) to be used as criteria for eligibility for the review	☒	☐	97-117
**Information sources**	9	Describe all intended information sources (e.g., electronic databases, contact with study authors, trial registers, or other grey literature sources) with planned dates of coverage	☒	☐	132-142
**Search strategy**	10	Present draft of search strategy to be used for at least one electronic database, including planned limits, such that it could be repeated	☒	☐	132-142
STUDY RECORDS					
Data management	11a	Describe the mechanism(s) that will be used to manage records and data throughout the review	☒	☐	154- 160
Selection process	11b	State the process that will be used for selecting studies (e.g., two independent reviewers) through each phase of the review (i.e., screening, eligibility, and inclusion in meta-analysis)	☒	☐	136- 138
Data collection process	11c	Describe planned method of extracting data from reports (e.g., piloting forms, done independently, in duplicate), any processes for obtaining and confirming data from investigators	☒	☐	144- 153
**Data items**	12	List and define all variables for which data will be sought (e.g., PICO items, funding sources), any pre-planned data assumptions and simplifications	☒	☐	176- 219
**Outcomes and prioritization**	13	List and define all outcomes for which data will be sought, including prioritization of main and additional outcomes, with rationale	☒	☐	239-283
**Risk of bias in individual studies**	14	Describe anticipated methods for assessing risk of bias of individual studies, including whether this will be done at the outcome or study level, or both; state how this information will be used in data synthesis	☒	☐	336- 340
DATA					
**Synthesis**	15a	Describe criteria under which study data will be quantitatively synthesized	☒	☐	176- 219
	15b	If data are appropriate for quantitative synthesis, describe planned summary measures, methods of handling data, and methods of combining data from studies, including any planned exploration of consistency (e.g., I^2, Kendall's tau)	☒	☐	220-323
	15c	Describe any proposed additional analyses (e.g., sensitivity or subgroup analyses, meta-regression)	☒	☐	324-335
	15d	If quantitative synthesis is not appropriate, describe the type of summary planned	☒	☐	341-344
**Meta-bias(es)**	16	Specify any planned assessment of meta-bias(es) (e.g., publication bias across studies, selective reporting within studies)	☒	☐	340
**Confidence in cumulative evidence**	17	Describe how the strength of the body of evidence will be assessed (e.g., GRADE)	☒	☐	337-340

For peer review only

BioMed Central
The Open Access Publisher

BMJ Open

The effect of dose on the antimalarial efficacy of artesunate-mefloquine against Plasmodium falciparum malaria: a protocol for systematic review and individual patient data (IPD) meta-analysis

Journal:	*BMJ Open*
Manuscript ID	bmjopen-2018-027738.R1
Article Type:	Protocol
Date Submitted by the Author:	22-Mar-2019

Complete List of Authors:	Mansoor, Rashid; WorldWide Antimalarial Resistance Network (WWARN); University of Oxford, Centre for Tropical Medicine and Global Health, Nuffield Department of Medicine<br>Dahal, Prabin; WorldWide Antimalarial Resistance Network (WWARN); University of Oxford, Centre for Tropical Medicine and Global Health, Nuffield Department of Medicine<br>Humphreys, Georgina; Wellcome Trust<br>Guerin, Philippe; WorldWide Antimalarial Resistance Network (WWARN); University of Oxford, Centre for Tropical Medicine and Global Health, Nuffield Department of Medicine<br>Ashley, Elizabeth ; Myanmar-Oxford Clinical Research Unit (MOCRU), Yangon, Myanmar; University of Oxford, Centre for Tropical Medicine and Global Health, Nuffield Department of Medicine<br>Stepniewska, Kasia; WorldWide Antimalarial Resistance Network (WWARN); University of Oxford, Centre for Tropical Disease and Global Health, Nuffield Department of Medicine

**Primary Subject Heading**:	Infectious diseases
Secondary Subject Heading:	Epidemiology, Public health
Keywords:	malaria, Plasmodium falciparum, efficacy, IPD meta-analysis, mefloquine, artemisinin

SCHOLARONE™ Manuscripts

For peer review only - http://bmjopen.bmj.com/site/about/guidelines.xhtml

The effect of dose on the antimalarial efficacy of artesunate-mefloquine against *Plasmodium falciparum* malaria: a protocol for systematic review and individual patient data (IPD) meta-analysis

Rashid Mansoor[1,2]†, Prabin Dahal[1,2]†, Georgina S Humphreys[3], Philippe J Guerin[1,2], Elizabeth A Ashley[2,4], Kasia Stepniewska[1,2]*

[1]WorldWide Antimalarial Resistance Network (WWARN), Oxford, UK

[2]Centre for Tropical Medicine and Global Health, Nuffield Department of Clinical Medicine, University of Oxford, Oxford, UK

[3]Wellcome Trust, London, UK

[4]Myanmar Oxford Clinical Research Unit, Yangon, Myanmar

Running title: dose effect of artesunate mefloquine

† contributed equally

*Corresponding author

Dr. Kasia Stepniewska: kasia.stepniewska@wwarn.org

Address: Centre for Tropical Medicine and Global Health, Nuffield Department of Medicine Research Building, University of Oxford, Old Road Campus, Roosevelt Drive, Oxford OX3 7FZ Tel: +44-01865 612900

Email:

rashid.mansoor@wwarn.org (RM);

prabin.dahal@ndm.ox.ac.uk (PD);

g.humphreys@wellcome.ac.uk (GSH);

philippe.guerin@wwarn.org (PJG);

liz@tropmedres.ac (EAA);

kasia.stepniewska@wwarn.org (KS)

Word count 3776

For peer review only - http://bmjopen.bmj.com/site/about/guidelines.xhtml

Abstract

Introduction: Antimalarial posology based on weight-bands leave patients at the margins vulnerable to receiving either lower or higher weight-adjusted (mg/kg) dosages. This article aims to describe the protocol for systematic review and individual patient meta-analysis for a study of the distribution of artesunate and mefloquine dosage administered in patients with uncomplicated *P. falciparum* malaria treated with an artesunate-mefloquine (AS-MQ) regimen. Relationship between mg/kg dose and therapeutic outcomes will be assessed through a one-stage individual participant data (IPD) meta-analysis (MA).

Methods and analysis: Therapeutic efficacy studies with the AS-MQ regimen will be identified by searching the following databases: PUBMED, EMBASE, and Web of Science. The corresponding authors of the relevant studies will be requested to share the IPD for the purpose of this meta-analysis to a secured repository. All available studies will be standardised using a common data management protocol and pooled into a single database. The relationship between mg/kg dosage and treatment failures will be assessed using a Cox regression model with study sites considered as a shared frailty term. Safety parameters will be explored where available.

Ethics and Dissemination: This IPD meta-analysis met the criteria for waiver of ethical review as defined by the Oxford Tropical Research Ethics Committee (OxTREC) as the research consists of secondary analysis of existing anonymous data. The results of this analysis will be disseminated at conferences, WWARN website and any peer-reviewed publication arising will be made open-source.

PROSPERO Registration: CRD42018103776

Keywords: malaria, *Plasmodium falciparum*, efficacy, IPD meta-analysis, mefloquine, artemisinin, artemisinin combination therapy

For peer review only

For peer review only - http://bmjopen.bmj.com/site/about/guidelines.xhtml

Strengths and limitations of this study

- With the exception of recent studies in Southeast Asia, the regimen artesunate-mefloquine has consistently demonstrated an efficacy greater than 95% and treatment failures in any single antimalarial study are few. This IPD-MA will allow a robust exploration of host, parasite and drug factors associated with therapeutic outcomes, which otherwise would not be possible.
- The proposed IPD-MA will allow exploration of variations in the weight-adjusted dosage received by patients, which is not possible with aggregate data meta-analysis.
- The IPD-MA will be carried out as a study group under the auspices of the WorldWide Antimalarial Resistance Network (WWARN), which has championed responsible data sharing and advocates translational research.
- A limitation of this analysis will be heterogeneity between studies included in terms of design, patient population and the susceptibility of the parasites against the drug regimen.

For peer review only - http://bmjopen.bmj.com/site/about/guidelines.xhtml

Introduction

The combination artesunate-mefloquine (AS-MQ) was the first antimalarial regimen developed as an Artemisinin Combination Therapy (ACT) when mefloquine resistance became rampant along the Thai-Myanmar border in the early 1990s [1]. The efficacy of a combination regimen (artemisinin derivative + partner component) depends upon the ability of the partner component to mop up the residual parasites leftover after the initial and highly potent anti-parasitic activity of the artemisinin derivatives. This requires the dosage of the partner drug to be sufficient to ensure that blood concentrations exceed the Minimum Inhibitory Concentration (MIC) of the parasites until all the parasites have been killed. Manufacturers' recommendations regarding antimalarial posology are often pragmatic and the dose is administered based upon weight "banding". This approach inevitably results in some patients at the band margins receiving either lower or higher dosages when adjusted for body weight (Figure 1). Young children are particularly vulnerable to extreme total dosages especially when drug administration is based on tablets rather than paediatric formulations or suspensions. This may lead to sub-therapeutic drug concentrations in the blood plasma and such under-exposure has been related to poorer therapeutic response for some of the widely used ACTs [2–4].

Until recently ACTs have consistently demonstrated an efficacy greater than 95% and treatment failures in any single antimalarial study are few thus limiting the ability to draw inferences regarding putative factors associated with therapeutic outcomes. Individual participant data (IPD) meta-analysis (MA) is being used increasingly to explore some of the putative factors which otherwise would not be possible through aggregate data meta-analysis[5]. Such a IPD-MA approach has been used to assess the dose-response relationships for the ACT regimens of artesunate-amodiaquine, artemether-lumefantrine and dihydroartemisinin-piperaquine [2,3,6]. These studies have demonstrated that the drug formulation (fixed vs loose) and under-exposure in the paediatric population due to weight-banding are deterministic of

For peer review only - http://bmjopen.bmj.com/site/about/guidelines.xhtml

poorer therapeutic outcomes. A thorough evaluation of the dose-response relationship for the regimen AS-MQ is lacking and this IPD-MA aims to address this research gap.

Objectives

The overall aim of this study is to determine the mg/kg dosing range of artesunate (AS) and mefloquine (MQ) adopted in clinical trials and to investigate the effects of mg/kg dosing on clinical outcome.

The specific objectives are:

- To investigate the effects of mefloquine and artesunate mg/kg dosing on early and late clinical outcomes (treatment success or failure)
- To investigate the tolerability of AS-MQ across different study sites, population and age groups

For peer review only

For peer review only - http://bmjopen.bmj.com/site/about/guidelines.xhtml

Methods and analyses

Criteria for study eligibility

Studies identified will be deemed eligible for the purpose of this analysis if they meet the following criteria:

- Prospective clinical efficacy study (defined as a trial which enrolled patients with confirmed diagnosis of malaria and who were follow-up for at least 28 days post-treatment) of uncomplicated *P. falciparum* (either alone or mixed infections) in patients of all ages
- Assessing the efficacy of a fixed-dose AS-MQ combination, either as single tablet type, or co-blister pack of more than one tablet type, or assessing the efficacy of a loose combination of AS-MQ
- Where AS was given over three days (with any number of doses per day) with a target total dose of 6-30mg/kg
- Where MQ was given over 1-3 days, on any of days 1-3 (with any number of doses per day) with a target total dose of 15-33mg/kg
- Where all AS and MQ were administered orally
- With a minimum of 28 days follow up
- With genotyping performed for late parasite recurrence
- With individual patient data on dosage of mefloquine received (actual or per protocol) by patients (dosage per tablets, number of tablets given per dose and duration of treatment)

Criteria for study exclusion

• Where other antimalarial drugs were given in addition to the initial ASMQ treatment regimen, except for a single dose of primaquine of 0.25mg/kg in the first 3 days

For peer review only - http://bmjopen.bmj.com/site/about/guidelines.xhtml

Types of study participants

Patients with uncomplicated *P. falciparum* malaria will be included in this IPD-MA. The following patients will be excluded from the analysis:

i. Severe *P. falciparum* malaria
ii. Pregnant women

Types of intervention/exposure and controls

- Fixed dose combination of AS-MQ, either as single tablet type, or co-blister pack of more than one tablet type, or loose combination of AS-MQ. AS given over three days (with any number of doses per day) with a target total dose of 6-30mg/kg. MQ given over 1-3 days, on any of days 1-3 (with any number of doses per day) with a target total dose of 15-33mg/kg

Types of outcomes

i. Parasitological and clinical efficacy
ii. Adverse events

Information sources and search strategy

We will carry out a systematic review and search PubMed, EMBASE, and Web of Science to identify publications with artesunate-mefloquine between Jan 1990 to July 2018, the full search terms are available from the PROSPERO registration (CRD42018103776) and also presented as supplementary file (Supplementary File 1). We do not plan to search grey literature for the purpose of this review.

Any important protocol amendments will be documented in the PROSPERO registration. Studies will be included regardless of language and publication status. Study screenings will be carried by two independent reviewers who will screen title, abstract, full text as necessary.

For peer review only - http://bmjopen.bmj.com/site/about/guidelines.xhtml

The following studies will be excluded: animal models (e.g. mouse malarias *P. berghei, P. chabaudi*), publications only including severe malaria, studies with follow up <28 days, data previously included in another published study, prophylaxis or mass drug administration studies, studies in healthy volunteers/challenge studies, or studies in asymptomatic patients or pregnant women.

Data acquisition and data management

Collating IPD

Principal investigators of the published (or unpublished) studies identified from the literature search will be invited to share IPD. At least three emails will be sent out in case of non-response. Researchers agreeing to the terms and conditions of the submission will be requested to upload anonymised IPD to the WWARN repository through a secure web portal. Figure 2 shows the process map which depicts the different phases of data procurement; data acquisition from the contributors, data standardisation, and their subsequent re-use in IPD meta-analysis.

Data will be fully anonymised and handled in compliance with the UK Data Protection Act to protect personal information and patient privacy. Original data will be stored on a secure server hosted by the University of Oxford.

Data management

Raw data from individual studies will be standardised using an open and transparent data management and statistical analysis protocol[7]. Investigators will be further contacted for validation or clarification, if required, and individual study protocols will be requested. On standardisation, the data will be stored in a relational database of several tables containing information on drug regimen, parasitological, clinical, and haematological assessments, genotyping and therapeutic outcomes; all linked by a unique patient identifier.

For peer review only

For peer review only - http://bmjopen.bmj.com/site/about/guidelines.xhtml

Data contributors participation

All the researchers who share individual patient data from eligible studies will become part of the study group; will have an opportunity to contribute to the analysis, interpretation of the results, manuscript preparation; and will be listed as co-authors on the publication arising from these analyses according to the WWARN publication policy.

Statistical analysis plan

Study Population

The following patients will be included in the analysis:

i. Information is available on drug dosage, either as exact number of tablets received, exact mg/kg dose received or number of tablets planned per protocol

ii. Date of the last day of follow-up or length of follow-up

The following patients will be excluded from the analysis:

i. Received other antimalarial drugs during follow-up before recorded *P. falciparum* treatment failure

ii. Results of genotyping performed for late parasitological outcome are not available

iii. Missing confirmation of *P. falciparum* infection on enrolment

iv. Missing age or weight or gender

v. Other deviations, as defined in the data management plan[7] :

(a) Haemoglobin < 5 g/dL on day 0

(b) Haematocrit < 15% on day 0

For peer review only

Outcomes

Primary: PCR-corrected *P. falciparum* recrudescence

For peer review only - http://bmjopen.bmj.com/site/about/guidelines.xhtml

195 Secondary: PCR-corrected *P. falciparum* reinfection

196 PCR-uncorrected *P. falciparum* recurrence

197 Early parasitological responses on days 1, 2 and 3

198 Gametocyte carriage within 14 days of treatment initiation in patients without
199 gametocytaemia at enrolment

200 Anaemia status on day 7

201 Adverse symptoms developed after the drug administration

202 The primary endpoint for this IPD-MA is Polymerase Chain Reaction (PCR) genotyping
203 corrected risk of *P. falciparum* recrudescence (treatment failure) on day 42. The day 42 was
204 selected as the primary endpoint based on the current recommendations from the WHO as
205 outlined in the 2009 protocol, which suggests that day 42 be the minimum follow-up period
206 for the mefloquine regimen [8]. In the analysis of the primary endpoint, patients in whom new
207 infections are observed during the study follow-up, or those who are lost to follow-up will be
208 censored; the former on the day new infection was observed and the latter on their last recorded
209 visit day. For the analysis of PCR corrected new infections, patients with recrudescence and
210 those who are lost to follow-up will be censored. Further definitions of status and other
211 censorship are detailed in the Clinical Module DMSAP [7].

212 Acute drug vomiting within an hour of treatment administration, general vomiting within 7
213 days of treatment initiation, diarrhoea within 7 days of treatment initiation, and,
214 neuropsychiatric adverse events (where available) are secondary endpoints.

For peer review only - http://bmjopen.bmj.com/site/about/guidelines.xhtml

Variables and definition

Artesunate and mefloquine doses received will be calculated from the number of tablets administered to each patient daily. If the actual number of tablets received is not recorded, the total dose in mg or mg/kg recorded as administered to each patient will be used. If none of these are available, administration as per protocol will be assumed. The current recommended daily mefloquine dose is 8.3 (range: 5 - 11) mg/kg [9]. A patient will be classified as under-dosed if the 3-day total mg/kg mefloquine dose is less than 15 mg/kg.

Nutritional status in children under 5 years of age will be assessed using standardised age, weight, height and gender specific growth reference standards according to the WHO 2006 recommendations using igrowup Stata package [10]. Anthropometric indicators include weight-for-age (WAZ), height-for-age (HAZ), and weight-for-height (WHZ). The nutritional status of a child will be given as a Z-score and classified as stunted, underweight or wasted as defined in the WHO guidelines.

The falciparum malaria transmission intensity of the study sites will be assessed using the prevalence estimates generated by the Malaria Atlas Project (MAP) based on the latitude, longitude and the year the study was conducted [11,12].

Anaemia will be defined as haemoglobin (Hb) < 10 g/dL or haematocrit (Ht) < 30%. Severe anaemia is defined as haemoglobin < 7 g/dL or haematocrit < 20%. Fever will be defined as body temperature > 37.5 degrees Celsius.

Parasite resistance status will be defined (data permitting) for each patient from South-East Asia region based on the reported prevalence of mutations of molecular markers (pfmdr1, kelch13) or the distribution of parasite clearance half-life for their study site and year of

For peer review only - http://bmjopen.bmj.com/site/about/guidelines.xhtml

admission [13,14]. For other locations, we will assume that parasites are sensitive to the ASMQ regimen.

Descriptive summary

Summary of the studies

Summary of included studies will be presented with respect to: study location, years of study, study population, duration of follow-up, AS-MQ drug formulation, methodology for parasite quantification, methodology for PCR genotyping, supervision of drug administration. PCR-corrected and uncorrected outcomes will be used to compute the Kaplan-Meier (K-M) estimates using the censoring rules outlined above. The K-M estimates will be presented graphically together with the number of patients in the risk set.

Summary of the patients

Summary of baseline characteristics of the patients included in the analysis will be presented for each study, by region and in overall. The following baseline characteristics of patients will be presented: age; weight, parasitaemia on enrolment; presence of fever (body temperature > 37.5 degrees Celsius); haemoglobin (or haematocrit); anaemia (Hb < 10 g/dL) or severe anaemia (Hb < 7 g/dL), gametocytes on presentation, description of infection (*P. falciparum* or mixed infections), total mg/kg dose for each drug component, dosing strategies (age-based, weight based etc.), dose formulation (fixed or loose etc.), and supervision of drug administration. The number of available patients will be summarised for all variables, proportion will be used for categorical or binary variables, and mean and standard deviation (or median and range) will be used for continuous variables.

Analysis of the primary endpoint

The efficacy estimates for each of the studies will be summarised using the Kaplan-Meier (K-M) method.

For peer review only - http://bmjopen.bmj.com/site/about/guidelines.xhtml

Cox regression analysis will be carried out to identify the predictors associated with parasitic recrudescence using a one-stage IPD-MA. Random effects in the form of shared gamma frailty parameters will be used to adjust for study-site effect and account for unobserved statistical heterogeneity [15]. Schoenfeld residuals against transformed time will be used to determine if the assumption of proportional hazard is met. Cox-Snell residuals will be examined to determine the appropriateness of model fit. Martingale residuals will be used to assess the functional form of the covariates. Potential non-linear relationships between continuous variable and the treatment outcomes will be investigated using multivariable fractional polynomials (FP)[16]. If the assumption of proportional hazards is not satisfied, alternative approaches such as piecewise proportional hazards model, interaction with time, stratifying by the variable for which the assumption isn't satisfied or flexible parametric models will be considered. Variable selection process will follow a procedure described below.

Analyses of secondary endpoints

***P. falciparum* new infection:** The analysis of new infections will be similar to the analysis of the primary endpoint.

Parasite clearance: Early parasitological responses will be assessed by the parasite positivity rate (PPR), which is the proportion of patients remaining parasitaemic on days 1, 2 and 3 post treatment administration[17]. The relationship between mg/kg dosage of the artesunate and mefloquine on early parasitological responses will be explored using a logistic regression model with study sites fitted as random effect. Variable selection and additional sensitivity analyses will follow the plan as outlined for the primary endpoint.

Gametocyte carriage: Gametocyte carriage during the study follow-up will be stratified by the gametocytaemia status at baseline. For those with documented gametocytaemia at enrolment, proportion of patients in whom gametocyte has cleared will be reported. For those

For peer review only - http://bmjopen.bmj.com/site/about/guidelines.xhtml

285 without gametocytes on enrolment, the proportion of patients in whom gametocytes have evolved will be presented. The relationship between mg/kg dosage of the artesunate and mefloquine on gametocyte endpoints will be explored using a logistic regression model with study sites fitted as random effect. Variable selection and additional sensitivity analyses will follow the plan as outlined for the primary endpoint.

290 **Haematological insult:** Anaemia during the study follow-up will be stratified by the anaemia status at baseline. For those who are anaemic at enrolment (, the proportion of patients who have recovered will be reported. For those who are not anaemic at enrolment, the proportion of patients whom are subsequently anaemic will be presented. The relationship between mg/kg dosage of the artesunate and mefloquine on anaemia endpoints will be explored using a logistic regression model with study sites fitted as random effect. Variable selection and additional sensitivity analyses will follow the plan as outlined for the primary endpoint.

297 **Safety endpoints:** The proportion of patients with acute drug vomiting, vomiting and diarrhoea, neuropsychiatric adverse events within a week of treatment initiation will be reported. The relationship between the mg/kg mefloquine dose and safety endpoints will be evaluated using a logistic regression model with study sites fitted as a random effect, if data permits. Variable selection and additional sensitivity analyses will follow the plan as outlined for the primary endpoint.

For peer review only - http://bmjopen.bmj.com/site/about/guidelines.xhtml

Variable selection strategy

The following covariates will be examined: age, sex, weight, baseline parasitaemia (except for new infection analysis), weight-for-age z-score (WAZ), underweight for age termed underweight (defined as WAZ < -2), haemoglobin, gametocytes on presentation (except for new infection analysis), past history of malaria (if available); description of infection: mixed species infections (except for new infection analysis), presence of markers of drug resistance e.g. kelch13 mutations or *pfmdr1* amplification (if available), details of treatment received: total mg/kg dose of AS and MQ, regimen, drug supervision, and vomiting of medication. Year of enrolment will also be included to account for changes in parasite susceptibility over time.

A general strategy recommended by Collet (2015)[18] will be followed for the construction of multivariable regression model:

i) All possible risk factors will be examined in a univariable analysis. The log-likelihood estimates ($-2 \times Log\hat{L}$) will be compared against the null model to assess if any of the variables reduce its value at 5% level of statistical significance.

ii) All the variables identified in step (i) will be fitted together in one model and variables which are not significant in the presence of other variables based on the results of the Wald test will be identified.

iii) A likelihood ratio test will be used to assess the impact of omitting variables identified in step (ii). If the omitted variable does not significantly impact the model log-likelihood, then they will be dropped. Only those variables which lead to significant change in log-likelihood are retained.

iv) All variables excluded from step (i) will be added to the model identified in step (iii) one by one to check if they provide any improvement to the model.

v) A final check of the model identified in step (iv) will be carried out to ensure that none of the variables in the model can be omitted without significantly increasing the model

For peer review only - http://bmjopen.bmj.com/site/about/guidelines.xhtml

log-likelihood, and none of the excluded variables significantly reduces the model log-likelihood.

Comparison of likelihood between nested models will be conducted using Likelihood Ratio Test (LRT). Akaike's Information Criterion (AIC) will be used to compare non-nested models. Treatment dosage, drug formulation, and baseline parasitaemia will be included in the multivariable model as *a priori* forced variables regardless of their statistical significance. Variables with more than 50% observations missing will not be included in multivariable analysis. Interactions will be assessed between dosing and the following variables: region, age group, transmission intensity, hyperparasitaemia (parasitaemia>100,000 parasites per microlitre), date of enrollment.

Assessment of statistical heterogeneity

The multilevel logistic or Cox models will be used for explaining study-site heterogeneity. Heterogeneity across study sites will be assessed by the variance of the shared frailty term estimated in the random effect Cox model or variance of the random intercepts in logistic regression. Additionally, intra-class correlation in logistic regression model will be reported.

Subgroup analyses

Analyses will be conducted by geographical region, drug regimen and resistance status if data permit.

Sensitivity analyses

A model will be refitted with each study's data excluded, one at a time, and a coefficient of variation around the parameter estimates will be calculated. This would identify any influential studies, that is, studies with unusual results (due to variations in methodology, patient population, and so on) that affect the overall pooled analysis findings. To assess the impact of missing data (covariates, PCR genotyping results), sensitivity analysis will be performed to see

For peer review only - http://bmjopen.bmj.com/site/about/guidelines.xhtml

if our main conclusion is affected or not by the exclusion of patients with missing data. Multiple Imputation (MI) will be used for handling missing data for missing covariates and missing outcomes. MI will be carried out for covariates with missing proportion less than 50%. [19]

Quality assessment/risk of bias assessment in studies included

Two reviewers will independently assess risk of bias. The risk of bias within and across the studies included in the analysis will be carried out using the GRADE guidelines[20]. Cochrane risk of bias tool 2.0 will be used to assess risk of bias in individual randomised controlled trails. Publication bias will assessed by a funnel plot[21].

Assessment of risk of potential bias in missing studies

Despite best possible efforts, it is anticipated that raw data from all the identified studies will not be available. Risk of potential bias in these studies will be assessed using a two-stage IPD-MA for the reported efficacy outcomes.

For peer review only

For peer review only - http://bmjopen.bmj.com/site/about/guidelines.xhtml

Further development of statistical analysis plan

The main analysis is planned as described above. Modification or additional analyses may be required as the data collection progresses. An updated statistical analysis plan will be available on the WWARN study group website[22].

Software

All statistical analyses will be carried out using R (The R Foundation for Statistical Computing) or Stata (4905 Lakeway Drive, College Station, Texas 77845 USA). Using alternative statistical software will not require amendment of this SAP.

Ethics and dissemination

This IPD meta-analysis met the criteria for waiver of ethical review as defined by the Oxford Tropical Research Ethics Committee (OxTREC) as the research consists of secondary analysis of existing anonymous data[23]. All studies included in this analysis will have received local ethical approvals and our pooled IPD meta-analysis will be addressing scientific questions that are very similar to the original research questions.

Findings will be reported following the PRISMA-IPD guidelines[24] at open access peer-reviewed journals. This systematic literature review and IPD meta-analysis is registered to PROSPERO (CRD42018103776) and this protocol has been reported following the PRISMA-P guidelines[25]. Any publications based on the findings of this IPD meta-analysis will be in accordance with the guidelines of the International Committee of Medical Journal Editors.

Patient and Public Involvement

Patients were not involved in the development of the research question, outcome measure or study design.

For peer review only

For peer review only - http://bmjopen.bmj.com/site/about/guidelines.xhtml

Discussion

Large scale deployment of highly efficacious ACT regimens such as artesunate-mefloquine has been the cornerstone of global malaria control for over a decade and this has contributed to the global reduction of mortality and morbidity associated with malaria [12]. Maintaining these gains is highly dependent on efficient health systems, sustainable global funding and the current status of antimalarial drug resistance. The 2017 WHO report found that globally the number of malaria cases has stopped dropping and mortality has crept up compared to 2016, suggesting that the recent public health gains remain fragile [26,27]. To make things worse, a health calamity is looming large due to the emergence of resistance to artemisinins in South-East Asia which is threatening to reverse the remarkable progress achieved over the past decade [28]. In the absence of an alternative treatment class to replace the ACTs as first line therapy, preserving the currently available drugs remains the top-most priority and this requires the highest form of evidence regarding the susceptibility of the parasites against the antimalarial drugs. Artesunate-mefloquine retains extremely high efficacy in most locations except Thailand, and in any given trial, only few treatment failures have been observed which limits the utility of any single study in answering questions regarding the dose-response relationship. Individual participant data (IPD) meta-analysis provides an alternative strategy.

IPD meta-analysis is now considered the gold-standard for evidence synthesis and allows exploration of different risk factors which otherwise would not be possible through the aggregate data meta-analysis [5]. This IPD-MA is designed to explore the variability in drug dosage administered in patients with uncomplicated *P. falciparum* malaria, treated with artesunate-mefloquine (AS-MQ). The WHO-recommended AS-MQ regimen is administered as a three day course, with a total of 12 mg/kg artesunate and 25 mg/kg of mefloquine split over three days. Due to the poor tolerability of high dose mefloquine, the dose of mefloquine is usually divided into either two doses (15 and 10 mg/kg), or three as a fixed dose combination

For peer review only - http://bmjopen.bmj.com/site/about/guidelines.xhtml

(8mg/kg/day). The fixed dose combination has been shown to provide better efficacy and improve treatment adherence for artesunate-amodiaquine [6]. Such a comparison is yet to be made for the AS-MQ regimen, and in this IPD-MA we propose to compare the fixed and loose formulations of the regimen with regards to the drug dosing, tolerability, efficacy and practicality of the dose banding.

In conclusion, this pooled analysis will provide critical information regarding the relationship between drug dosage and parasitological responses post-treatment with artesunate-mefloquine. The assessment of the host, parasite and drug determinants that influence the treatment response can provide evidence-based guidance for monitoring the early signs of artemisinin resistance and effective case management that will be critical in optimising malaria control and containment efforts.

For peer review only

For peer review only - http://bmjopen.bmj.com/site/about/guidelines.xhtml

Declarations

Author contributions

Conceived the idea and wrote the first draft of the protocol: RM, PD, PG, EAA, and KS

Systematic review of all published antimalarial studies: GSH

Data acquisition and standardisation: GSH, EAA, KS

All authors read and approved the submission of the final draft of the study protocol.

Funding

The WorldWide Antimalarial Resistance Network (RM, PD, GSH, RB, PJG, and KS) is funded by a Bill and Melinda Gates Foundation grant and the ExxonMobil Foundation. The funders did not participate in the study development, the writing of the paper, decision to publish, or preparation of the manuscript.

Competing interests

The authors declare that they have no competing interests.

Ethics approval

This individual patient data meta-analysis met the criteria for waiver of ethical review as defined by the Oxford Tropical Research Ethics Committee (OxTREC) since the research consists of secondary analysis of existing anonymous data. Each study included in the analysis will have received local ethics approvals.

Acknowledgements

We would like to thank Dr. Makoto Saito for his several helpful suggestions. We would like to thank Brittany Maguire for her help with the manuscript.

For peer review only - http://bmjopen.bmj.com/site/about/guidelines.xhtml

List of Figures

Figure 1: Mg/kg dose variations for mefloquine

Legend: The WHO recommendation on the 3-day fixed dose AS-MQ regimen is to administer once daily 50 mg of MQ for those weighing 5-8 kg, 100 mg for 9-17 kg, 200 mg for 18-29 kg and 400mg for those who weigh ≥ 30 kg. The horizontal dotted line represents the target MQ dose of 25 mg/kg. Since the dosing is based on weight-bands, this leads to the saw-tooth dosing curves.

Figure 2: Data acquisition and standardisation process utilizing the WWARN database

Legend: Individual participant data is shared by researchers to the WWARN secured portal (left panel). The studies are standardised using a common WWARN protocol and the tables of clinical, parasitological and drug measurements are stored in a secured repository with relational database (middle panel). On standardisation, the dataset is shared back with the study investigator, and subsequently used in meta-analyses to answer questions of public health importance (right panel).

For peer review only

For peer review only - http://bmjopen.bmj.com/site/about/guidelines.xhtml

461 ## References

462 1. Nosten, F. *et al.* Treatment of multidrug-resistant Plasmodium falciparum malaria with 3- day artesunate-mefloquine combination. *J. Infect. Dis.* **170,** 971–977 (1994).

464 2. The WorldWide Antimalarial Resistance Network (WWARN) DP Study Group. The Effect of Dosing Regimens on the Antimalarial Efficacy of Dihydroartemisinin-Piperaquine: A Pooled Analysis of Individual Patient Data. *PLoS Med.* **10,** 1–17 (2013).

468 3. WorldWide Antimalarial Resistance Network (WWARN) Lumefantrine PK/PD Study Group. Artemether-lumefantrine treatment of uncomplicated Plasmodium falciparum malaria: a systematic review and meta-analysis of day 7 lumefantrine concentrations and therapeutic response using individual patient data. *BMC Med.* **13,** 227 (2015).

472 4. Hoglund, R. M. *et al.* Population Pharmacokinetic Properties of Piperaquine in Falciparum Malaria: An Individual Participant Data Meta-Analysis. *PLoS Med.* **14,** 1–23 (2017).

475 5. Riley, R. D., Lambert, P. C. & Abo-Zaid, G. Meta-analysis of individual participant data: Rationale, conduct, and reporting. *BMJ* **340,** 521–525 (2010).

477 6. The WorldWide Antimalarial Resistance Network (WWARN) AS-AQ Study Group. The effect of dosing strategies on the therapeutic efficacy of artesunate-amodiaquine for uncomplicated malaria: a meta-analysis of individual patient data. *BMC Med.* **13,** 66 (2015).

481 7. WorldWide Antimalarial Resistance Network. Data Management and Statistical Analysis Plan Version 1.2. *www.wwarn.org* (2012). Available at: http://www.wwarn.org/sites/default/files/ClinicalDMSAP.pdf. (Accessed: 24th

For peer review only

For peer review only - http://bmjopen.bmj.com/site/about/guidelines.xhtml

September 2018)

8. World Health Organization. *Methods for Surveillance of Antimalarial Drug Efficacy*. (2009).

9. World Health Organization. *Guidelines for the treatment of malaria: Third edition*. (2015).

10. WHO Multicentre Growth Reference Study Group. WHO Child Growth Standards: Length/height-for-age, weight-for-age, weight-for-length, weight-for-height and body mass index-for-age: Methods and development. . *Geneva World Heal. Organ.* 312 (2006).

11. Gething, P. W. *et al.* A new world malaria map: Plasmodium falciparum endemicity in 2010. *Malar. J.* **10,** 378 (2011).

12. Bhatt, S. *et al.* The effect of malaria control on *Plasmodium falciparum* in Africa between 2000 and 2015. *Nature* 1–9 (2015). doi:10.1038/nature15535

13. WWARN Parasite Clearance Study Group. Baseline data of parasite clearance in patients with falciparum malaria treated with an artemisinin derivative: an individual patient data meta-analysis. *Malar. J.* **14,** 359 (2015).

14. WWARN. WWARN Molecular Surveyor. *www.wwarn.org* (2018). Available at: http://www.wwarn.org/molecularsurveyor/. (Accessed: 22nd October 2018)

15. Glidden, D. V & Vittinghoff, E. Modelling clustered survival data from multicentre clinical trials. *Stat. Med.* **23,** 369–88 (2004).

16. Royston, P. & Sauerbrei, W. in *Multivariable Model-Building: A Pragmatic Approach to Regression Analysis based on Fractional Polynomials for Modelling Continuous Variables* pp: 71-98 (John Wiley & Sons, Ltd, 2008).

For peer review only - http://bmjopen.bmj.com/site/about/guidelines.xhtml

17. WWARN ACT Africa Baseline Study Group. Clinical determinants of early parasitological response to ACTs in African patients with uncomplicated falciparum malaria: a literature review and meta-analysis of individual patient data. *BMC Med.* **13,** 212 (2015).

18. Collett, D. in *Modelling survival data in medical research* pp: 83-87 (CRC Press, 2015).

19. White, I. R., Royston, P. & Wood, A. M. Multiple imputation using chained equations: Issues and guidance for practice. *Stat. Med.* **30,** 377–399 (2011).

20. Guyatt, G. *et al.* GRADE guidelines: 1. Introduction - GRADE evidence profiles and summary of findings tables. *J. Clin. Epidemiol.* 383–394 (2011). doi:10.1016/j.jclinepi.2010.04.026

21. Sedgwick, P. Meta-analyses: How to read a funnel plot. *BMJ* **346,** 1–2 (2013).

22. www.wwarn.org. The WWARN AS-MQ Dose Impact Study Group. (2018). Available at: http://www.wwarn.org/working-together/study-groups/mq-dose-impact-study-group. (Accessed: 24th September 2018)

23. OxTREC. Oxford Tropical Research Ethics Committee. (2018). Available at: https://researchsupport.admin.ox.ac.uk/governance/ethics/committees/oxtrec. (Accessed: 24th September 2018)

24. Stewart, L. A. *et al.* Preferred reporting items for a systematic review and meta-analysis of individual participant data: The PRISMA-IPD statement. *J. Am. Med. Assoc.* **313,** 1657–1665 (2015).

25. Shamseer, L. *et al.* Preferred reporting items for systematic review and meta-analysis protocols (prisma-p) 2015: Elaboration and explanation. *BMJ* **349,** 1–25 (2015).

For peer review only

For peer review only - http://bmjopen.bmj.com/site/about/guidelines.xhtml

530 26. World Health Organization. *World Malaria Report 2017*. (2017).

531 27. World Health Organization. *World Malaria Report 2016*. (2016).

532 28. Ashley, E. A. *et al.* Spread of Artemisinin Resistance in Plasmodium falciparum
533 Malaria. *N. Engl. J. Med.* **371,** 411–423 (2014).

534

For peer review only - http://bmjopen.bmj.com/site/about/guidelines.xhtml

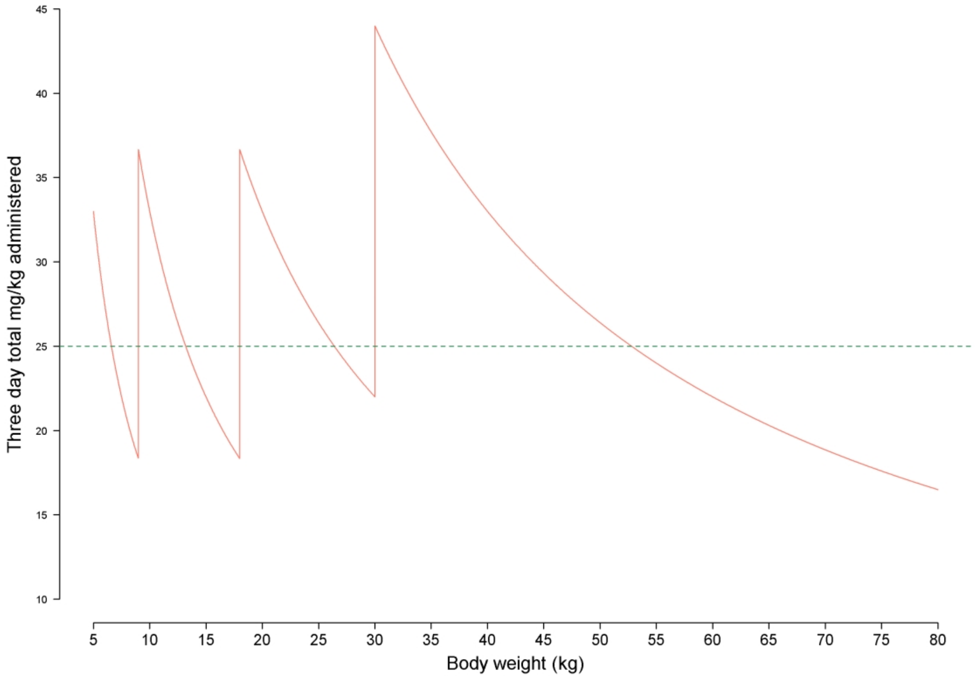

Caption : Figure 1:Mg/kg dose variations for mefloquine
Legend: The WHO recommendation on the 3-day fixed dose AS-MQ regimen is to administer once daily 50 mg of MQ for those weighing 5-8 kg, 100 mg for 9-17 kg, 200 mg for 18-29 kg and 400mg for those who weigh ≥ 30 kg. The horizontal dotted line represents the target MQ dose of 25 mg/kg. Since the dosing is based on weight-bands, this leads to the saw-tooth dosing curves.

239x179mm (300 x 300 DPI)

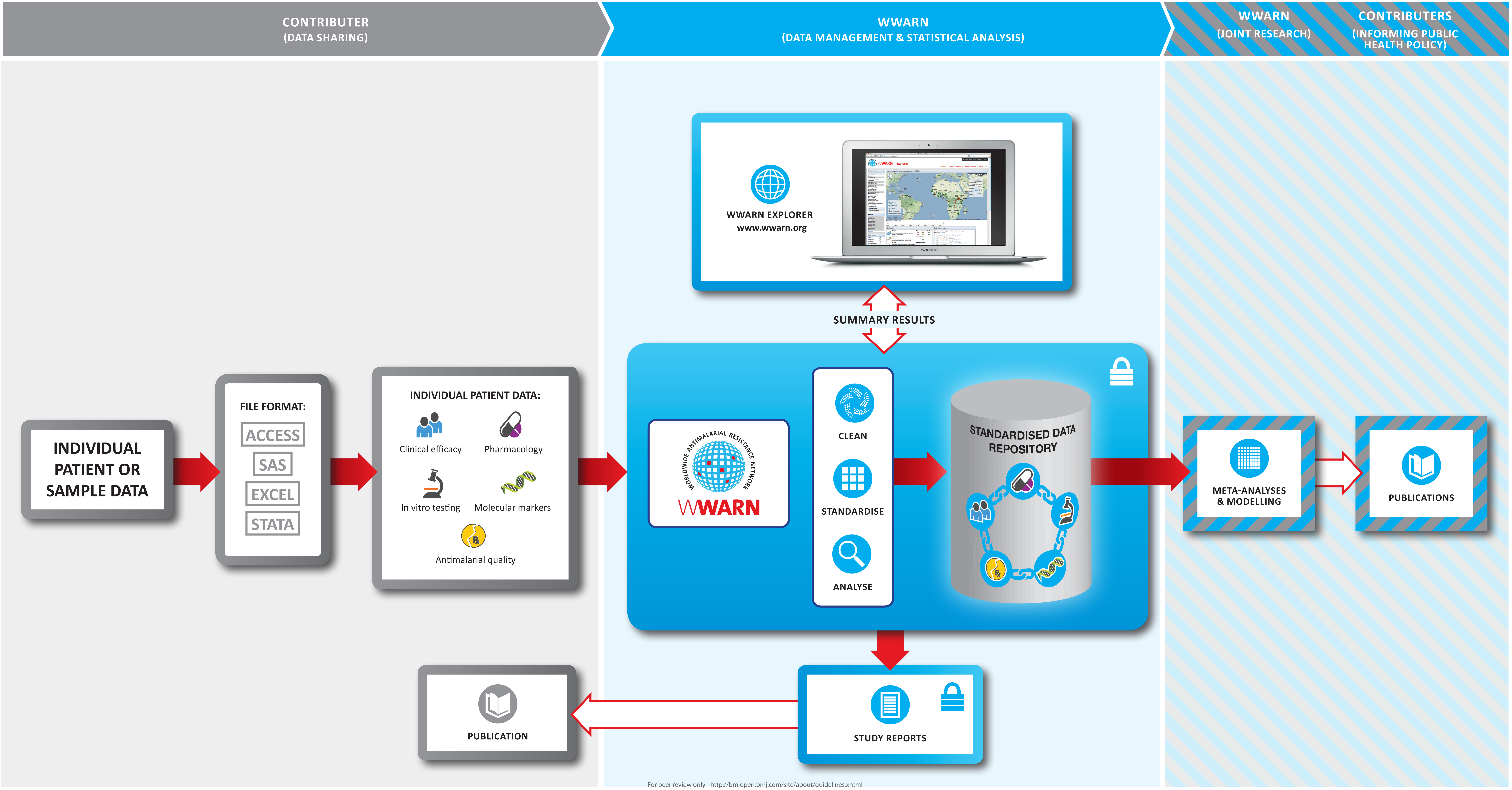
CONTRIBUTER
(DATA SHARING)
WWARN
(DATA MANAGEMENT & STATISTICAL ANALYSIS)
WWARN
(JOINT RESEARCH)
CONTRIBUTERS
(INFORMING PUBLIC HEALTH POLICY)
WWARN EXPLORER
www.wwarn.org
SUMMARY RESULTS
INDIVIDUAL PATIENT OR SAMPLE DATA
FILE FORMAT:
ACCESS
SAS
EXCEL
STATA
INDIVIDUAL PATIENT DATA:
Clinical efficacy
Pharmacology
In vitro testing
Molecular markers
Antimalarial quality
WORLDWIDE ANTIMALARIAL RESISTANCE NETWORK
WWARN
CLEAN
STANDARDISE
ANALYSE
STANDARDISED DATA REPOSITORY
META-ANALYSES & MODELLING
PUBLICATIONS
STUDY REPORTS
PUBLICATION

Supplementary file: The effect of dose on the antimalarial efficacy of artesunate-mefloquine against *Plasmodium falciparum* malaria: a protocol for systematic review and individual patient data (IPD) meta-analysis

Rashid Mansoor[1,2], Prabin Dahal[1,2], Georgina S Humphreys[3], Philippe J Guerin[1,2], Elizabeth A Ashley[2,4], Kasia Stepniewska[1,2]*

[1]WorldWide Antimalarial Resistance Network (WWARN), Oxford, UK

[2]Centre for Tropical Medicine and Global Health, Nuffield Department of Clinical Medicine, University of Oxford, Oxford, UK

[3]Wellcome Trust, London, UK

[4]Myanmar Oxford Clinical Research Unit, Yangon, Myanmar

*Corresponding author

Dr. Kasia Stepniewska

Centre for Tropical Medicine and Global Health,

Nuffield Department of Medicine Research Building,

University of Oxford, Old Road Campus,

Roosevelt Drive, Oxford OX3 7FZ

Tel: +44-01865 612900

Email : kasia.stepniewska@wwarn.org

For peer review only

Literature Search Strategy

The following search terminologies were used for searching relevant publications in the given libraries

PubMed via PubMed

((("plasmodium falciparum"[MeSH Terms] OR ("plasmodium"[All Fields] AND "falciparum"[All Fields]) OR "plasmodium falciparum"[All Fields]) OR falciparum[All Fields]) OR ("malaria"[MeSH Terms] OR "malaria"[All Fields])) AND (((((artesunate-mefloquine[All Fields] OR artesunate/mefloquine[All Fields]) OR ASMQ[All Fields]) OR AS-MQ[All Fields]) OR AS/MQ[All Fields]) OR (("artesunate"[Supplementary Concept] OR "artesunate"[All Fields]) AND (("mefloquine"[MeSH Terms] OR "mefloquine"[All Fields]) OR MQ[All Fields])))

EMBASE via OVID

(plasmodium falciparum.mp. OR Plasmodium falciparum/ OR falciparum.mp. OR (falciparum.mp. AND (Plasmodium/ OR plasmodium.mp.)) OR malaria.mp. OR malaria/) AND (artesunatemefloquine.mp. OR ASMQ.mp. OR AS-MQ.mp. OR ((artesunate/ OR artesunate.mp.) AND (mefloquine/ OR mefloquine.mp. OR MQ.mp.))

BIOSIS via Web of Science

(TS=(plasmodium falciparum) OR (TS=(falciparum AND plasmodium)) OR TS=(falciparum) OR TS=(malaria)) AND TS=(artesunate-mefloquine) OR TS=(ASMQ) OR TS=(AS-MQ) OR (TS=(artesunate) AND TS=(mefloquine OR MQ))

Web of Science Core Collection via Web of Science

(TS=(plasmodium falciparum) OR (TS=(falciparum AND plasmodium)) OR TS=(falciparum) OR TS=(malaria)) AND TS=(artesunate-mefloquine) OR TS=(ASMQ) OR TS=(AS-MQ) OR (TS=(artesunate) AND TS=(mefloquine OR MQ))

For peer review only

PRISMA-P 2015 Checklist

This checklist has been adapted for use with protocol submissions to *Systematic Reviews* from Table 3 in Moher D et al: Preferred reporting items for systematic review and meta-analysis protocols (PRISMA-P) 2015 statement. *Systematic Reviews* 2015 **4**:1

Section/topic	#	Checklist item	Information reported: Yes	Information reported: No	Line number(s)
**ADMINISTRATIVE INFORMATION**					
**Title**					
Identification	1a	Identify the report as a protocol of a systematic review	☒	☐	2-3
Update	1b	If the protocol is for an update of a previous systematic review, identify as such	☐	☒	NA
**Registration**	2	If registered, provide the name of the registry (e.g., PROSPERO) and registration number in the Abstract	☒	☐	44
**Authors**					
Contact	3a	Provide name, institutional affiliation, and e-mail address of all protocol authors; provide physical mailing address of corresponding author	☒	☐	5-22
Contributions	3b	Describe contributions of protocol authors and identify the guarantor of the review	☒	☐	402- 406
**Amendments**	4	If the protocol represents an amendment of a previously completed or published protocol, identify as such and list changes; otherwise, state plan for documenting important protocol amendments	☐	☒	NA
**Support**					
Sources	5a	Indicate sources of financial or other support for the review	☒	☐	407- 411
Sponsor	5b	Provide name for the review funder and/or sponsor	☒	☐	409
Role of sponsor/funder	5c	Describe roles of funder(s), sponsor(s), and/or institution(s), if any, in developing the protocol	☒	☐	409-411
**INTRODUCTION**					
**Rationale**	6	Describe the rationale for the review in the context of what is already known	☒	☐	61- 86
**Objectives**	7	Provide an explicit statement of the question(s) the review will address with reference to participants, interventions, comparators, and outcomes (PICO)	☒	☐	87- 95
**METHODS**					

BioMed Central The Open Access Publisher

2

Section/topic	#	Checklist item	Information reported Yes	Information reported No	Line number(s)
**Eligibility criteria**	8	Specify the study characteristics (e.g., PICO, study design, setting, time frame) and report characteristics (e.g., years considered, language, publication status) to be used as criteria for eligibility for the review	☒	☐	97-117
**Information sources**	9	Describe all intended information sources (e.g., electronic databases, contact with study authors, trial registers, or other grey literature sources) with planned dates of coverage	☒	☐	132-142
**Search strategy**	10	Present draft of search strategy to be used for at least one electronic database, including planned limits, such that it could be repeated	☒	☐	132-142
STUDY RECORDS					
Data management	11a	Describe the mechanism(s) that will be used to manage records and data throughout the review	☒	☐	154- 160
Selection process	11b	State the process that will be used for selecting studies (e.g., two independent reviewers) through each phase of the review (i.e., screening, eligibility, and inclusion in meta-analysis)	☒	☐	136- 138
Data collection process	11c	Describe planned method of extracting data from reports (e.g., piloting forms, done independently, in duplicate), any processes for obtaining and confirming data from investigators	☒	☐	144- 153
**Data items**	12	List and define all variables for which data will be sought (e.g., PICO items, funding sources), any pre-planned data assumptions and simplifications	☒	☐	176- 219
**Outcomes and prioritization**	13	List and define all outcomes for which data will be sought, including prioritization of main and additional outcomes, with rationale	☒	☐	239-283
**Risk of bias in individual studies**	14	Describe anticipated methods for assessing risk of bias of individual studies, including whether this will be done at the outcome or study level, or both; state how this information will be used in data synthesis	☒	☐	336- 340
DATA					
**Synthesis**	15a	Describe criteria under which study data will be quantitatively synthesized	☒	☐	176- 219
	15b	If data are appropriate for quantitative synthesis, describe planned summary measures, methods of handling data, and methods of combining data from studies, including any planned exploration of consistency (e.g., I^2, Kendall's tau)	☒	☐	220-323
	15c	Describe any proposed additional analyses (e.g., sensitivity or subgroup analyses, meta-regression)	☒	☐	324-335
	15d	If quantitative synthesis is not appropriate, describe the type of summary planned	☒	☐	341-344
**Meta-bias(es)**	16	Specify any planned assessment of meta-bias(es) (e.g., publication bias across studies, selective reporting within studies)	☒	☐	340
**Confidence in cumulative evidence**	17	Describe how the strength of the body of evidence will be assessed (e.g., GRADE)	☒	☐	337-340

BioMed Central
The Open Access Publisher
